# Application of Enhanced T1WI of MRI Radiomics in Glioma Grading

**DOI:** 10.1155/2022/3252574

**Published:** 2022-05-13

**Authors:** Hongzhang Zhou, Rong Xu, Haitao Mei, Ling Zhang, Qiyun Yu, Rong Liu, Bing Fan

**Affiliations:** ^1^Medical College of Nanchang University, Nanchang 330036, China; ^2^Jiangxi Provincial People's Hospital, Nanchang 330006, China; ^3^The First Affiliated Hospital of Nanchang Medical College, Nanchang 330006, China

## Abstract

**Objective:**

To explore the application value of the radiomics method based on enhanced T1WI in glioma grading.

**Materials and Methods:**

A retrospective analysis was performed using data of 114 patients with glioma, which was confirmed using surgery and pathological tests, at our hospital between January 2017 and November 2020. The patients were randomly divided into the training and test groups in a ratio of 7 : 3. The Analysis Kit (AK) software was used for radiomic analysis, and a total of 461 tumor texture features were extracted. Spearman correlation analysis and the least absolute shrinkage and selection (LASSO) algorithm were employed to perform feature dimensionality reduction on the training group. A radiomics model was then constructed for glioma grading, and the validation group was used for verification.

**Results:**

The area under the ROC curve (AUC) of the proposed model was calculated to identify its performance in the training group, which was 0.95 (95% CI = 0.905–0.994), accuracy was 84.8%, sensitivity was 100%, and specificity was 77.8%. The AUC of the validation group was 0.952 (95% CI = 0.871–1.000), accuracy was 93.9%, sensitivity was 90.0%, and specificity was 95.6%.

**Conclusions:**

The radiomics model based on enhanced T1WI improved the accuracy of glioma grading and better assisted clinical decision-making.

## 1. Introduction

Glioma is the most common primary tumor of the human brain, which accounts for about 30%–40% of the human central nervous tumors. The WHO suggested classifying gliomas into four grades. It is generally believed that grades I and II are low-grade gliomas (LGGs) and grades III and IV are high-grade gliomas (HGGs). There are major differences in the prognosis and treatment of gliomas of different grades. Surgical resection is the preferred treatment for LGGs, and adjuvant radiotherapy and chemotherapy [1, 2] are required for treating HGGs postoperatively. Studies have shown that the higher the tumor grade, the higher is the postoperative recurrence rate and the lower is the patient's survival rate [3]. Therefore, accurate grading of gliomas before surgery is essential to guide the selection of treatment and improve the prognosis of the patients. Magnetic resonance imaging (MRI) is the most commonly used examination method for preoperative diagnosis of glioma. Although functional MRI and multiparameter imaging have made rapid progress in the diagnosis and identification of glioma in recent years, they have shown limited accuracy in tumor grading before the surgery [4]. Additionally, due to individual differences in patients, gliomas show significant heterogeneity [5, 6]. Although traditional imaging methods are very useful, they cannot fully meet the needs of precision medicine [7].

Radiomics was proposed by Lambin in 2012 [8] to extract and analyze a large number of quantitative image features in a high-throughput manner [8]. Previously, radiomics has been applied in tumor studies, including meningioma and lung cancer [9, 10]. The current study reports the application of MRI radiomics based on enhanced T1WI to establish a convenient and noninvasive method to grade glioma before surgery and improve the accuracy of diagnosis.

## 2. Data and Methods

### 2.1. Patients' Information

The current research was approved by the Ethics Committee of the Jiangxi Provincial People's Hospital (No. 2022–026). Since it was a retrospective study, procuring informed consent from the patients was exempted. A retrospective analysis was performed using data of patients who were admitted to our hospital between January 2017 and November 2020 and met the following criteria. Inclusion criteria were as follows: MRI showed intracranial space-occupying lesions, which were later confirmed by surgery and pathological tests as glioma, and the pathological grade was clear; and the intracranial lesions were not treated (using, for example, surgery, chemotherapy, radiotherapy, biopsy, or hormone therapy). Exclusion criteria were as follows: the case information was incomplete or cannot be followed up for any reason and due to motion or magnetic-sensitive artifacts, the image quality was poor and could not meet the research requirements.

Among the 134 glioma patients, 10 received preoperative chemotherapy, 2 had a history of surgery, and 8 patients were lost to follow-up due to various reasons. Therefore, data of 114 patients were considered, 35 LGG (WHO grade II) cases and 79 HGG (21 cases of WHO grade III and 58 cases of grade IV) cases. Surgery was performed on all the patients included in the study 1-2 weeks after the MRI images were collected. The pathological diagnosis was performed by a neuropathologist having 20 years of clinical experience.

### 2.2. Collection of MRI Images

The 3.0 MRI scanner (GE Discovery MR750 W) and an 8-channel head-phased array coil were used. The sequences used were head axial routine plain scan T1WI, T2WI, and contrast-enhanced T1WI sequence. Gadolinium-containing contrast agent (0.1 mL/kg Ga-DTPA; Bayer, Germany) was injected at a rate of 2.0 mL/s. After injecting 20 mL of saline at the same rate, the parameters of the T1WI sequence used were TR, 550 ms; TE, 12 ms; NEX 2, matrix 256 × 320; layer thickness, 5 mm; and field of view (Fov), 24 × 24 cm. The parameters of the T2WI used were TR, 5500 ms; TE, 100 ms; NEX 2, matrix 256 × 320; layer thickness, 5 mm; and Fov, 24 × 24 cm. The same machine and scan parameters were used to acquire images of all patients to minimize errors.

### 2.3. Image Analysis

Multiparameter MRI data were analyzed and processed on AW 4.6 workstation (Function Tool; GE Healthcare). The patients' gender and age were recorded. Two radiologists who had more than 8 years of experience used the blinding method to evaluate the features of glioma in the MR images, mainly the size of the lesion (measuring the maximum diameter of the tumor thrice and considering the average), proportion of tumors with a clear boundary, proportion of tumors with hemorrhage, and proportion of tumors with peritumoral edema. When the evaluation was inconsistent, a consensus was reached after discussing it.

### 2.4. Feature Extraction

**Figure 1:**
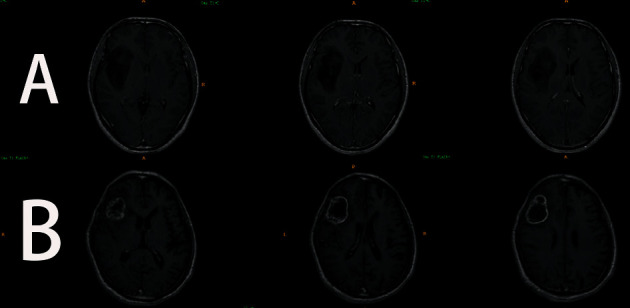
(a) LGG of the right temporal lobe (WHO grade II). (b) HGG of the right temporal lobe (WHO grade IV).

**Figure 2:**
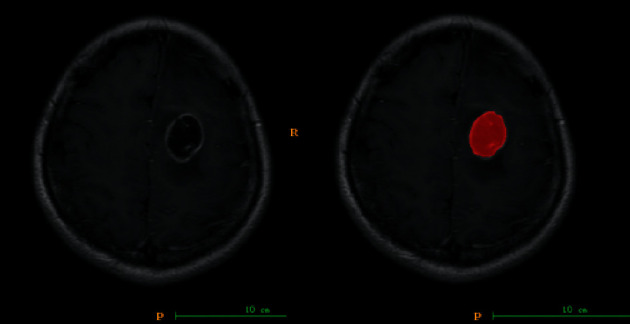
Using the image processing software (ITK-SNAP), the area of interest was manually outlined on all levels of the brain glioma (grade II) in the enhanced T1WI, and the levels merged into a 3D area of interest (red).

Images were imported into the image processing software (ITK-SNAP) in DICOM format, and the two radiologists, having no knowledge of the pathological results, using a semiautomatic method with an interactive level-set volume of interest via threshold-based and edge-based algorithms, outlined the region of interest (ROI) layer by layer on all levels of the displayed tumor in the enhanced T1WI sequences. To ensure the accuracy of the study, the ROIs should be selected as close as possible to the tumor boundary, while avoiding necrosis, calcification, and peritumoral edema [11]. When viewing the features of glioma in MRI images, the tumor boundary is the clearest in enhanced T1WI [12]. Therefore, all ROI images in this study were outlined in contrast-enhanced T1WI (Figures 1 and 2).

The original image and the segmented tumor ROI file were imported into the AK software at the same time (Artificial Intelligence Kit V3.0.0.R, GE Healthcare) [12, 13]. The software automatically extracted 461 quantitative image feature parameters including morphology, histogram, Haralick feature, run-length matrix (RLM), gray-level co-occurrence matrix (GLCM), and gray-level size zone matrix. Each feature value for the patients was obtained by performing normalization using a Z-score (x.m/s), where “*x*” represented the value of the feature, “*m*” represented the average value, and “*s*” represented the standard deviation. Before using the machine learning model for classification, the unit limit of each feature was eliminated.

### 2.5. Establishment of the Radiomics Model

Of the 114 patients, 80 comprised the training group and 34 comprised the test group, to maintain a ratio of 7 : 3. The redundancy between characteristic parameters was calculated using the Spearman method. If the correlation coefficient was greater than 90%, one of them was retained. Then, the least absolute shrinkage and selection (LASSO) regression algorithm was employed to reduce the dimensionality of the features [9, 13]. The logistic regression method was used to establish the prediction model using the selected parameters. Then, the selected radiomics features were linearly weighted according to the respective LASSO coefficients in the training and test groups to generate a radiomics risk score for the brain glioma grading.

### 2.6. Statistical Analysis

The “glmnet” package in the *R* software (3.6.1) was used to perform LASSO dimensionality reduction analysis. “glm” was used to build the regression model, and the “pROC” package was used to draw the ROC curve to evaluate the accuracy of the model. SPSS 23.0 software was used to perform statistical analysis using population age, gender, and MRI image features of the patients.

## 3. Results

### 3.1. Clinical Data and MRI Routine Imaging Features

There was no significant difference in the gender of patients between the LGG and HGG groups, but there was a significant difference in age. When comparing the imaging features between the two groups, the size of the tumor, proportion of tumors with a clear boundary, proportion of tumors with hemorrhage, and proportion of tumors with peritumoral edema were not significantly different. However, the enhancement of the tumor was significantly different between the two groups (Table 1).

### 3.2. Establishment of Radiomics Labels

**Figure 3:**
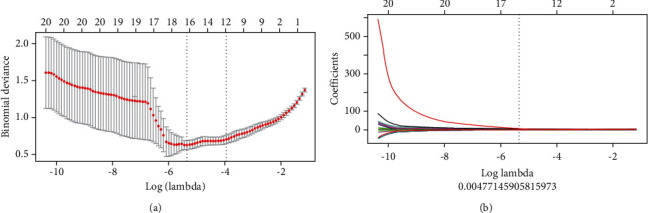
Screening radiomics features using LASSO regression. (a) LASSO regression uses cross-validation. The vertical dashed line on the left represents the log (*λ*) value corresponding to the best *λ* value. The selection standard was the minimum deviation value, i.e., −5.3. (b) The coefficients of texture parameters changing with *λ*. The vertical line corresponds to 9 features with nonzero coefficients selected using LASSO cross-validation.

The Analysis Kit (AK) was used to extract a total of 461 parameters from five categories, including morphological features, histogram features, gray-level co-occurrence matrix features, run-length matrix features, and gray-scale region size matrix features. Spearman correlation was used to calculate the redundancy between the feature parameters, and 0.9 was selected as the redundancy threshold. After deredundancy processing, 28 features were retained, and 9 feature parameters with a greater predictive value were selected using the LASSO dimensionality reduction algorithm (Figure 3, Table 2), including four histogram features, one Haralick feature, two GLCM features, one RLM feature, and one gray-level size zone matrix feature (Table 3). A linear equation was used to calculate the radiomics score for each patient, and the following formula was used:

Radscore = (2.951 × MeanDeviation) +  (−0.517 ×  quantile 0.025) + (0.141 × MedianIntensity) + (−0.488  ×  kurtosis) + (0.571 × Correlation_angle0_offset1)  + (0.541 ×  GLCMEntropy_AllDirection_offset4) + (4.399  ×  GLCMEntropy_AllDirection_offset7) + (0.757 ×  Long RunEmphasis_angle0_offset1) + (−1.02 ×  GrayLevel Nonuniformity_AllDirection_offset7).

**Figure 4:**
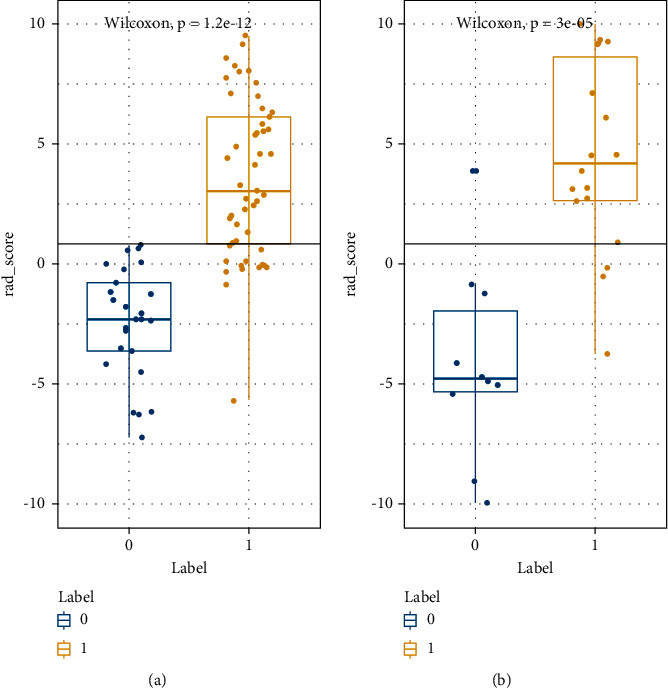
The comparison of LGG and HGG RAD scores in the training (a) and test groups (b). Labels 1 and 0 correspond to HGG and LGG, respectively.

**Figure 5:**
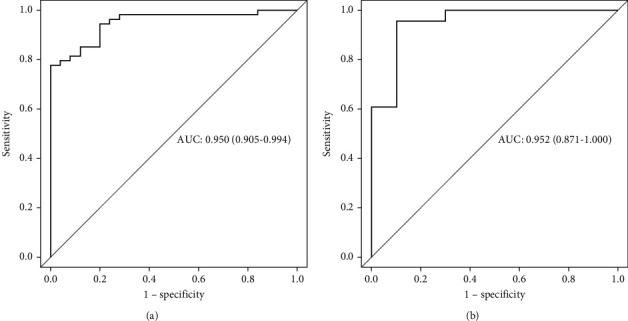
(a) For the training group (*n* = 80), the region of interest was used to evaluate the prediction model based on the enhanced T1WI, and the AUC value was 0.950. (b) According to the region of interest of the experimental group (*n* = 34), the prediction model based on the enhanced T1WI was evaluated, and the AUC value was 0.952.

The differences in RAD scores between HGG (label = 1) and LGG (label = 0) in the training and test groups were significantly different (*P* < 0.01) (Figure 4). The accuracy of the training and validation groups was 84.8% and 93.9%, respectively, the sensitivity was 100% and 90.0%, respectively, the specificity was 77.8% and 95.6%, respectively, and the AUC value was 0.950 and 0.952, respectively, suggesting the model showed good predictive performance in both the training and test groups (Figure 5 and Table 3).

## 4. Discussion

### 4.1. Development of Tumor ROI

In the current research, a radiomics model was structured for glioma grading by selecting 461 feature parameters. The model showed improved glioma grading. Building a predictive model requires extracting feature data, and delineating the region of interest is the prerequisite for extracting feature data. Most glioma tumors are rich in blood supply, and the enhancement is obvious in the enhanced T1WI sequences. Therefore, we chose to outline the region of interest in the enhanced T1WI sequences. Manual delineation is susceptible to other nonobjective factors. To reduce errors and obtain stable features, based on previous experience [9, 13, 14], the current study used two doctors for segmentation and the addition of interference noise.

### 4.2. Correlation of Texture Features with the Tumor

In the current study, 9 radiomics features were obtained using the enhanced T1WI. These features showed good predictive performance in the identification of LGG and HGG before surgery. There is complex heterogeneity in tumors, and conventional MR images show different signals and enhancement levels [15]. Since glioma is often accompanied by bleeding, the tumor signals are mixed due to intratumoral hemorrhage in different periods. However, the enhancement of the tumor still has a high degree of recognition, which is consistent with the results of glioma image features. Additionally, the heterogeneity is also manifested as different gray levels that are difficult to recognize using the naked eye [16], and histogram features and texture features can quantify the orientation, spatial distribution, and roughness of the texture in the tumor, which clearly and intuitively reflect the spatial heterogeneity of the different levels of glioma and provide more information for the preoperative graded diagnosis of glioma.

### 4.3. Application of the Radiomics Model in Glioma Grading

MRI is the most commonly used preoperative diagnostic method for glioma, but conventional MRI is often limited to positioning and auxiliary qualitative diagnosis and has little influence on glioma grading. Some progress has been made in MR functional imaging and multiparameter imaging diagnosis. However, the use of MRI for preoperative grading is still limited. Nowadays, radiomics methods are more and more widely used in the evaluation of various diseases. Some researchers have tried to apply it to meningioma, lung cancer, cervical cancer, and gastrointestinal stromal tumors [9, 10, 13, 14, 17]. Some researchers have also used similar methods to actively study glioma [18, 19]. Rathore collected 735 images of glioma patients and performed grading on these images, and the results showed that gliomas of different grades were different (accuracy = 0.751, AUC = 0.652) [20]. In the current study, we used software to extract high-throughput and multidimensional texture features. The results (accuracy = 0.848, AUC = 0.950) are better than those of the previous models, indicating that the proposed model is very reliable.

Nevertheless, the current research had certain limitations: the ROI of the current study was limited to the essence of the tumor, and there was insufficient research on peripheral necrosis and edema of the tumor. The sample size was considerably small, especially for low-grade brain glioma (there were no cases of grade I brain glioma and only 35 cases of grade II brain glioma). Therefore, even having found promising results, they need to be confirmed by futures studies with larger sample size. The study was a single-center retrospective study and lacked external verification using multicenter large sample data to ensure the reliability of the predictive model in actual clinical application.

## 5. Conclusions

In conclusion, the radiomics model based on enhanced T1WI imaging showed good prediction performance and has a certain guiding value for the presurgery grading prediction of brain glioma.

## Figures and Tables

**Table 1:** Clinical and imaging features of patients with LGG and HGG.

	LGG (*n* = 35)	HGG (*n* = 79)	Test value	*P* value
Gender (male/female)	18/17	42/37	0.029^a^	0.864
Age (year)	43 ± 10	60 ± 13	−7.122^t^	≤0.001^*∗*^
Max diameter (cm)	4.1 ± 1.3	3.9 ± 1.5	0.748^t^	0.456
Borders (indistinct/clear)	31/4	61/18	2.008^a^	0.156
Intratumoral bleeding (yes/no)	16/19	29/50	0.823^a^	0.364
Peritumoral edema (with/without or mild)	25/10	48/31	1.199^a^	0.274
The enhancement level			78.092^a^	≤0.001^*∗*^
No or light	26	3		
Moderate	9	11		
Severe	0	65		

*t*, *t*-test; ^a^chi square test.

**Table 2:** Texture parameters and their corresponding coefficient values after dimensionality reduction.

Feature parameter		Value
Histogram parameters	MeanDeviation	2.95
	Quantile 0.025	−0.51
	MedianIntensity	0.15
	Kurtosis	0.49

GLCM parameters	Correlation_angle0_offset1	0.53
	GLCMEntropy_AllDirection_offset4	0.51
	GLCMEntropy_AllDirection_offset7	4.45

RLM parameters	LongRunEmphasis_angle0_offset1	0.65
GLZSM parameters	GrayLevelNonuniformity_AllDirection_offset7	−1.01

GLCM, gray-level co-occurrence matrix; RLM, run-length matrix; GLZSM, gray-level size zone matrix.

**Table 3:** Diagnosis using the radiomics model in the training and test groups.

	AUC (95% CI)	Accuracy	Sensitivity	Specificity
Training group	0.950 (0.905–0.994)	0.848	1.0	0.778
Test group	0.952 (0.871–1.000)	0.939	0.9	0.956

## Data Availability

The datasets used and analyzed during the current study are available from the corresponding author upon request.
